# Diagnosis and Treatment for Pediatric Supracondylar Humerus Fractures with Brachial Artery Injuries

**DOI:** 10.3390/children8100933

**Published:** 2021-10-18

**Authors:** Tu Ngoc Vu, Son Hong Duy Phung, Long Hoang Vo, Uoc Huu Nguyen

**Affiliations:** 1 Department of Surgery, Hanoi Medical University, Hanoi 100000, Vietnam; vungoctu@hmu.edu.vn; 2 Department of Cardiovascular and Thoracic Surgery, Hanoi Medical University Hospital, Hanoi 100000, Vietnam; 3 Department of Cardiovascular and Thoracic Surgery, Viet Duc University Hospital, Hanoi 100000, Vietnam; 4 Institute for Preventive Medicine and Public Health, Hanoi Medical University, Hanoi 100000, Vietnam; vohoanglonghmu@gmail.com

**Keywords:** supracondylar humerus fracture, vascular injury, brachial artery injuries

## Abstract

(1) Background: This study aims to describe the clinical and paraclinical characteristics of and the diagnostic approach to brachial artery injuries in pediatric supracondylar humerus fractures, as well as to evaluate intraoperative vascular anatomical lesions and early postoperative results. (2) Methods: A retrospective, hospital-based analysis of medical records at Viet Duc University Hospital (Vietnam), using a sample of children under 16 years who met the diagnostic criteria for supracondylar humerus fractures with brachial artery injuries between January 2016 and December 2020, was performed. A total of 50 patients were included in the analysis. (3) Results: Out of 50 pediatric patients, 36 patients were male (72%) and the mean age was 5.85 years (range, 1.5–14 years). Before treatment, there were 46 patients with severely displaced fractures which were classified as Gartland type III (92%). Following casting, the percentage of those with severely displaced fractures was reduced significantly to 12%, while there were no patients with Gartland type III fractures after percutaneous pinning. Doppler sonography failed to assess vascular lesions at the fracture site before and after casting in most patients. Two-thirds of surgical cases had only vasospasm, without physical damage to the vessel wall or intravascular thrombosis. Preoperative Doppler spectrum analysis was not consistent with the severity of intraoperative brachial artery injury. Out of 24 patients with vasospasm, we performed vascular blockade using papaverin in 11 cases and intraoperative balloon angioplasty of the brachial artery using the Fogarty catheter in 13 cases. Brachial artery graft was performed with 12 patients who had anatomical damage to the vascular wall. A complication of embolism occurred in one patient immediately after surgery, and two patients had superficial infections. One month following surgery, 2 out of 36 patients had a temporary loss of sensation in the area of incision. (4) Conclusions: Most pediatric patients did not present with symptoms of critical limb ischemia similar to those associated with lower extremity vascular injuries. The diagnosis and treatment of pediatric supracondylar humerus fractures with vascular injury is difficult and time-consuming, especially in cases of transverse fractures.

## 1. Introduction

Supracondylar fractures of the humerus are the most prevalent kind of fractures, accounting for approximately 60% of all elbow fractures and 3–7% of pediatric fractures [1,2]. They are most common in young children between 5 and 10 years of age. More than 95% of these fractures are extension fractures, which may result in a variety of neurological and vascular complications. About 10% to 20% of displaced supracondylar fractures present alterations in vascular status [3,4]. An absent radial pulse was observed in 7% to 12% of all fractures and up to 19% in displaced fractures. Brachial artery injuries are often a consequence of stretching, entrapping or disrupting the neurovascular structures on the proximal fragment, as well as, though less frequently, of reduction maneuvers or immobilization of the elbow in the hyperflexion position [5,6].

The diagnosis and morphology of the anatomical lesion as well as the surgical treatment of this form of injury in children are markedly different from the extremity arterial injuries which are more common in adults. Through treating this pathology at our institution, we identified the following key concerns. Firstly, most brachial artery injuries in children do not have obvious symptoms of ischemia. Therefore, the implementation of intensive diagnostic procedures, as well as emergency surgery which is similar to that performed in cases of adult vascular trauma, can increase the risk of complications and consume unnecessary medical and social resources. Secondly, the application of Doppler ultrasound and multislice computed tomography angiography (CT-A) in children is not easy, especially for those who are not yet of school age. Anesthesia must be used in order to ensure the safety and effectiveness of these methods. As a result, the duration of the evaluation is prolonged and the pediatric patient may have to undergo anesthesia several times during the course of treatment, thereby increasing the risk of complications from anesthesia. Thirdly, once the vessel wall is opened for examination, the potential for endothelium lesion damage leads to actual occlusion of the artery because of the small diameter of the pediatric brachial artery. Once the vessel wall is opened for examination, the potential for endothelium lesion damage leads to actual occlusion of the artery because of the small diameter of the pediatric brachial artery. If the lesion is long, there is no alternative solution with artificial vessels or saphenous veins, as in adults. In addition, intervention on brachial arteries in pediatric patients has potential risks for adverse outcomes, such as vasodilation or vasoconstriction [7,8], large and bad scars [7,9] and osteomyelitis [10].

To better inform practice so as to minimize the duration of treatment and avoid the unnecessary consumption of medical resources, while ensuring that pediatric patients receive effective treatment for their injuries and avoid more complicated vascular complications, is the ultimate goal of this research. Our aims were to describe the clinical and paraclinical characteristics and the diagnostic approach to brachial artery injuries in pediatric supracondylar humerus fractures, and to evaluate intraoperative vascular anatomical lesions and early postoperative results.

## 2. Methods

### 2.1. Patients

This study was retrospectively performed using medical records at Viet Duc University Hospital, one of the oldest and largest surgical public hospitals in Vietnam, in the period from January 2016 to December 2020. We examined the data of patients who were under 16 years of age, had a diagnosis of traumatic arterial injury in the upper extremity and underwent treatment at our institution. Diagnostic criteria for supracondylar humerus fractures with brachial artery injuries included radiographs showing supracondylar humerus fractures and loss of the ulnar/radial pulse. We excluded those patients who had experienced previous elbow fracture(s) that caused limited movement and deformity.

### 2.2. Data Collection

Clinical parameters: age (years), age group (<3 years/3–<13 years/≥13 years), gender (male/female), injured side in the upper extremity (right/left), mechanism of injury (high energy trauma/other), types of bone fractures (closed fracture/open fracture), time interval between the beginning of trauma and arrival to the first health facility (hours), time interval between the beginning of trauma and arrival to the operating room (hours) and clinical symptoms.

Paraclinical parameters:

- X-ray: Gartland classification of fractures (type 1/type 2/type 3);
- Doppler sonography: vascular lesions at fracture site (thrombosis/crush injury/vasoconstriction), Doppler waveforms (monophasic flow/biphasic flow/triphasic flow) and flow velocity under the fracture site (m/s);
- Multislice CT-A for vascular injuries: short lesion, <5 mm/long lesion, ≥5 mm. Multislice CT-A was indicated for those with ischemic symptoms after unsuccesful conservative fracture treatment using casts.

Treatment:

- Conservative fracture treatment using casts;
- Vascular trauma treatment: intraoperative vascular blockade using papaverine/intraoperative balloon angioplasty of the brachial artery using the Fogarty catheter/Brachial artery graft by great saphenous vein;
- Early result and one-month reexamination were based on clinical assessment, elbow radiograph and Doppler sonography; handling complications included bone displacement and embolism.

### 2.3. Data Analysis

Data were sorted, cleaned, coded and entered into Epidata 3.1. Then, a software program (SPSS, version 23.0; IBM, Armonk, NY, USA) was used for all statistical analyses. Descriptive statistics, such as frequency, percentage, mean, standard deviation and interquartile range, were used to summarize preoperative, intraoperative and postoperative parameters.

## 3. Results

Among 50 pediatric patients, 36 patients were male (72%) and the mean age was 5.85 years (range, 1.5–14 years). The mean time interval between the beginning of trauma and arrival to the first health facility was 12 h (range, 1–120 h), and the mean time interval between the beginning of trauma and arrival to the operating room was 52.8 h (range, 4–168 h). Injuries were commonly the result of high energy trauma (*n* = 49, 98%). 31 patients injured their left arm (62.0%), while 19 injured their right arm (38.0%). Most patients were diagnosed with Gartland type III fractures (*n* = 46, 92%), the rest with Gartland type II fractures (*n* = 4, 8%). Pink hand was present in 49 patients with supracondylar fractures (98%), and purple hand in only 1 case (2%) (Table 1).

**Figure 1:**
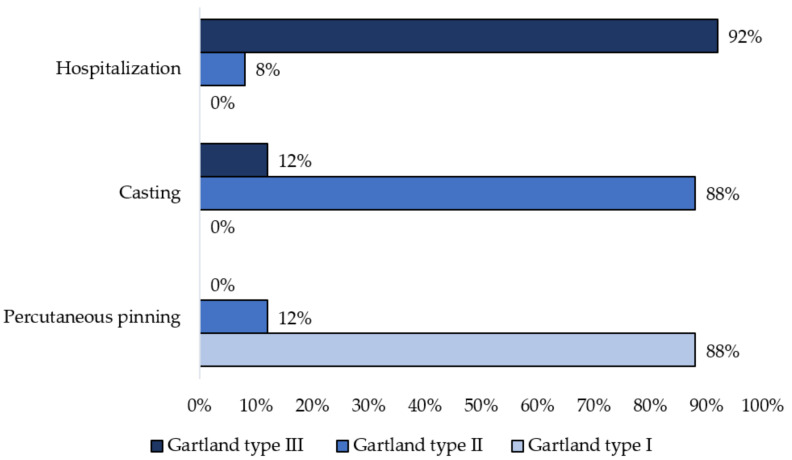
Changes of the Gartland classification of supracondylar humerus fractures after casting and after pinning.

Figure 1 indicates changes of the Gartland classification of supracondylar humerus fractures following casting and percutaneous pinning. Before treatment, there were 46 patients with severely displaced fractures; these were classified as Gartland type III (92%). Following casting, the percentage of those with severely displaced fractures decreased significantly to 12%, while there were no patients with Gartland type III fractures after pinning.

Doppler sonography failed to assess vascular lesions at the fracture site before and after casting in most patients (Table 2).

Out of 50 patients, all cases were firstly treated by casting, from them, 14 cases were successfully treated with a cast. Other 36 cases were indicated for surgery. As was shown in Table 3, all pediatric patients in the age group of 13 years or over intraoperatively had found vessel injury. Most lesions having length of ≥5 mm were indicated for surgery, while lesions < 5 mm were treated conservatively.

**Figure 2:**
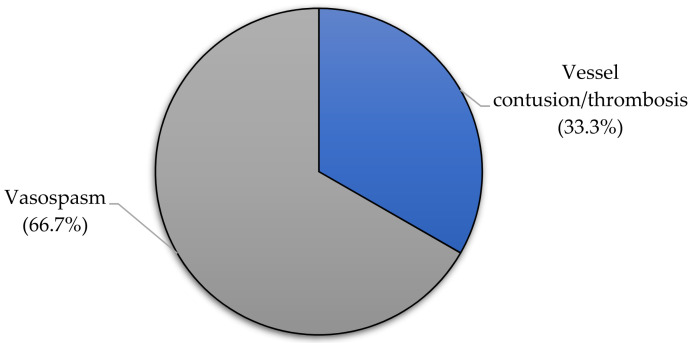
Intraoperative blood vessel injury in pediatric patients undergoing surgical treatment.

Two-thirds of surgical cases had only vasospasm without vessel wall contusion or intravascular thrombosis (*n* = 24, 66.7%) (Figure 2).

No patients under 3 years of age had anatomical damage of the vessel wall, while there were two patients over 13 years of age who were diagnosed with vessel wall contusion. Of the cases with long-segment lesions on multislice CT-A (≥5 mm) that were operated on, only one-third of the lesions were actually vascular contusion. Preoperative Doppler spectrum analysis was not consistent with the severity of intraoperative brachial artery injury. Out of 24 patients with vasospasm, we performed intraoperative vascular blockade using papaverin in 11 patients and intraoperative balloon angioplasty of the brachial artery using the Fogarty catheter in 13 patients. A brachial artery graft using great saphenous vein was performed in 12 patients with vascular wall contusion (Table 4).

As shown in Table 5, we recorded a complication of embolism occurring in one patient immediately after surgery and superficial infections in two patients. One month following surgery, 2 out of 36 patients experienced a temporary loss of sensation around an incision.

## 4. Discussion

### 4.1. Clinical Condition

Non-dominant hand injuries occur more frequently in pediatric insupracondylar humerus fractures. The incidence of fractures in men and women is almost equal [11,12]. Most fractures occurred in male children. The most common age is around 5–6 years old. This is the age when children are preparing or starting school and their awareness is still immature; it is difficult for them to control their movements and body postures, yet they are eager to learn and explore the world around them. 62% of children had fractures in the left hand, which is mostly non-dominant and less flexible compared to the right hand. Most were closed fractures due to high energy trauma, only a single case admitted to the hospital being an open fracture, caused by a buffalo ramming into the arm. The injury is mainly due to falling against the hand and the grounding distance is not too large, so all patients were in a stable condition when they were admitted to hospital.

Most of the patients had type III fractures based on the Gartland classification (92%). This is consistent with injury to the brachial artery in supracondylar humerus fractures, where the artery is trapped in the fracture. According to Pham Quang Tri [13], six out of eight patients with supracondylar humerus fractures with vascular injury had Gartland type III displacement. Most of the children who came to the hospital did not have obvious symptoms of ischemia. Only a few children showed cold extremities (22%) and cyanosis (2%), while none of them showed signs of ischemia or of severe or irreversible limb bleeding, which is common in adult limb vascular trauma. In our study, time intervals between the beginning of trauma and arrival at the first health facility, as well as arrival to the operating room, were much longer than the ideal time (6 h after the accident) to restore circulation after acute extremity embolism in adults. The prolonged time of hospital admission and delaying surgery in children could be explain by several reasons. First, the ischemia of the injury hand is not severe and it don’t influence on their condition. Second, diagnostic imaging procedures such as Doppler ultrasound, X-ray, and multislide CT-A, and casting procedure take more time among small children compared to among adults. Finally, the treatment algorism for this injury is casting firstly, after that, if the pulse is restored, we will keep stop on conservative treatment. If the pulse is not restored, we send to multislide CT-A and consider to surgery.

### 4.2. Preoperative Imaging

In the study of Phan Quang Tri [13], among 102 cases of supracondylar fractures that were treated with born reposition and percutaneous pinning under C-arm control. There were 8 cases of brachial artery injury, accounting for 7.48% in general and 57.2% of cases with complications. Because the clinical signs and symptoms of ischemia are not clear and difficult to find out in small children, especially those who are not yet of school age. Diagnostic imaging plays an important role in decision between surgical and conservative treatments. Most of the children being at school age, with little or no sense of cooperation, vascular imaging diagnostic procedures are very difficult to perform with high quality. The performance of vascular diagnostic tests requires anesthesia, leading to difficulties in deploying human resources and equipment, as well as carrying an increased risk of adverse consequences.

**Figure 3:**
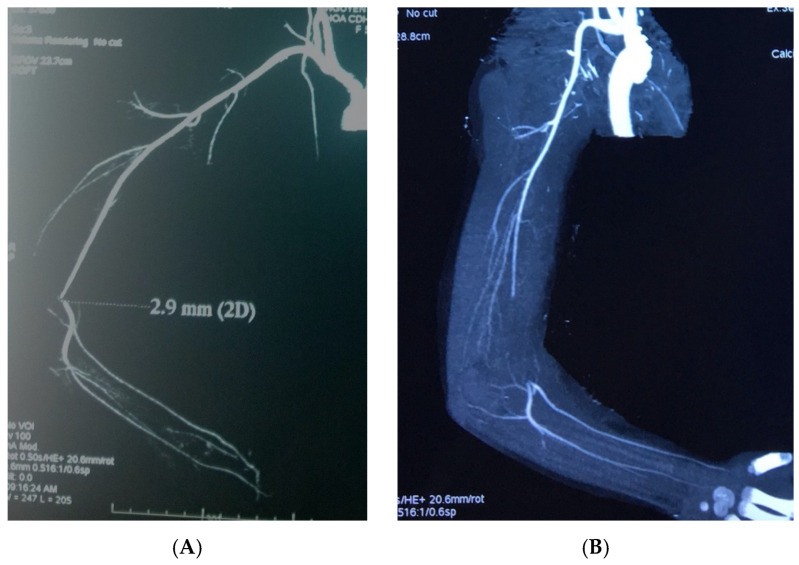
Brachial artery injuries in multislice CT-A. (**A**) Lesions < 5 mm. (**B**) Lesions ≥ 5 mm.

Because it is performed in emergency conditions with a lack of patient’s cooperation, cast, edema, and hematoma, only a very small number (<10%) of Doppler ultrasound analyses can directly evaluate the blood vessels at the level of the fracture, including the vessel wall and lumen. That information is quite needed for verify diagnosis of upper extremity vascular injury. In our study, more than half of the pediatric patients undergo a multislice CT-A of the upper extremity, whereas in adult patients these lesions are very rare to require this diagnostic procedure. Multislice CT-A enables an assessment of the perfusion status of the entire vascular system of the upper extremity but does not accurately assess the damage to the vessel wall and lumen in children. We found that all cases with length of lesion of the brachial artery on CT-A less than 5 mm did not necessarily require surgery. In addition, all study patients who had the lesions longer than 5 mm were indicated to surgery, but in only half of them can find out the vascular contusion intraoperatively. Therefore, multislice CT-A may be useful mainly in the decision of conservative therapy, i.e., those with short lesions (less than 5 mm) (Figure 3).

### 4.3. Efficacy of Vascular Rehabilitation after Treatment

For conventional closed supracondylar fractures of the humerus, treatment is achieved mainly by reposition of fractured born and cast or pinning. In cases with vascular injury, the good born reposition may enable to decompress blood vessels and restore blood flow. However, even with perfect born reposition, surgical treatment is still needed to treat the vascular injury in case having really anatomical damage to the vascular wall.

**Figure 4:**
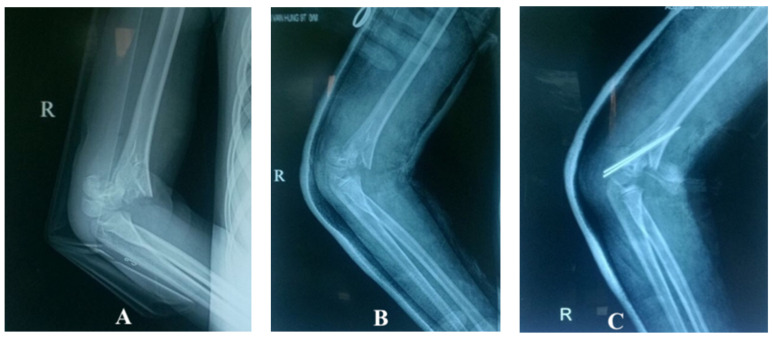
X-rays of supracondylar fractures of the humerus. (**A**) Before reposition—Gartland type III. (**B**) After reposition—Gartland type II. (**C**) After operative pinning—Gartland type II.

Among the techniques for achieving and maintaining born reposition, pinning is the most effective method, but it is also the most invasive and time-consuming, and is associated with risk of complications. By contrast, casts present the simplest and least invasive technique; they may not gave perfect reposition but could decompress vessel and restore blood flow. In this study, when patients were mainly diagnosed with type III displaced supracondylar fractures of the humerus before treatment (92%), this figure decreased significantly after cast to 12% and 0% after pinning (Figure 4).

### 4.4. Vascular Injury, Surgical Management and Related Factors

Regarding the cases of conservative treatment when born reposition and cast was applied but the radial pulse was still not found, if Doppler ultrasound analysis showed reduced blood flow and there were long-length of lesions on a multislice CT-A, patients were indicated for surgery with born reposition, pinning and re revascularization.

**Figure 5:**
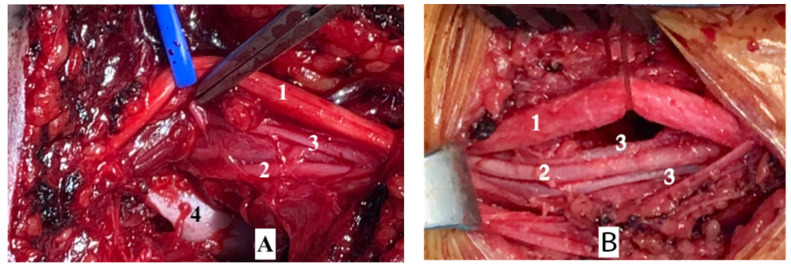
(**A**) Arterial entrapment in the fracture socket. (**B**) Freeing the artery from the fracture socket. (**1**: median nerve; **2**: brachial artery; **3**: brachial vein; **4**: fracture location.)

Intraoperatively found that, two-thirds of surgical cases had vasospasm without anatomical damage of the vessel wall or intravascular thrombosis. In most cases, following born reposition and cast, the blood vessels have been released from the fracture and only a few are still stuck in the fracture. All cases of surgery fractured born was reposited and fixed by pinning with K-wire from the lateral side. In cases with vasospasm. Blood flow will be restored by extra-vessel papaverine blockage or balloon dilatation of the brachial artery using a Fogarty catheter or cutting damaged artery and performing anastomosis end-to-end or replace it by great saphenous graft, while a brachial artery had wall damage (Figure 5).

**Figure 6:**
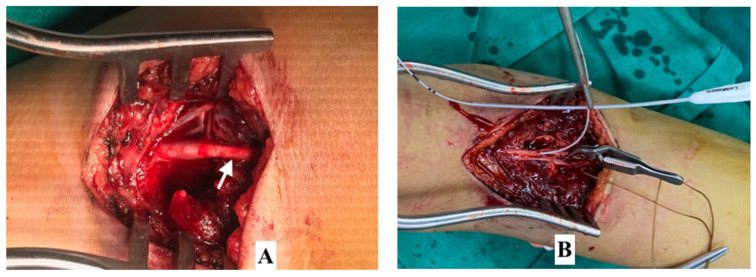
Vasospasm of brachial artery and its surgical management. (**A**) Vasospasm. (**B**) Balloon dilatation of the brachial ar-tery using the Fogarty catheter.

With vasospasm, there were several cases of short spasm which is not dilated after the pinning and blocking using papaverin. This opens the blood flow very weakly but without damage to the vessel wall and thrombosis of the lumen. This explains why on a multislice CT-A there can be seen a loss of the long brachial artery but no damage to the vessel wall during surgery. Therefore, if Doppler ultrasound accurately assesses the status of contusion in the transverse fracture and non-thrombotic lumen, angioplasty will most likely not need to be performed. This leads to a real vascular injury and the risk of a serious vascular complication, especially where a specialist in vascular surgery is not available (Figure 6).

**Figure 7:**
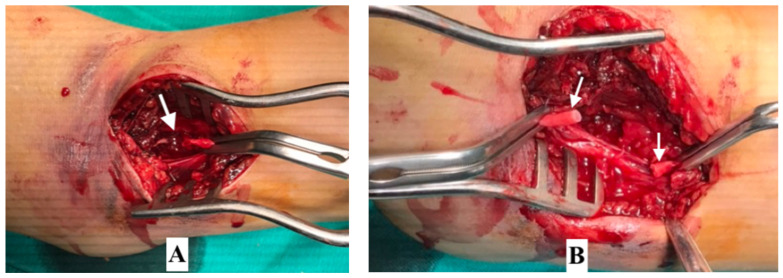
Physical injury to brachial artery and its surgical management. (**A**) Contusion, thrombosis and brachial artery nearly ruptured. (**B**) Damaged artery excised and directly anastomosed.

Comparing intraoperative finding of vascular lesions with the age group of the patient, we found that patients under 3 years of age only had vasospasm, while those over 13 years of age only had vascular contusion. This can be explained by ossification in the humerus bone. The ossification centers begin to fuse together at age 3 years and the ossification process completes by the age of 13 [14]. Therefore, in children less than 3 years of age, the bone structure is almost entirely cartilage which is not capable of causing damage to the vessel wall, while since the bone structure in children over 13 years old is almost adult, the pathology of vascular trauma is similar to that of an adult with typical vessel contusion and thrombosis. In the study, direct anastomosis end-to-end was performed when the lesions were short (less than 1 cm). In addition, the choice of grafting with saphenous vein is very difficult because of the very small size of vessel in children. However, in older children with sufficiently large vein size, the artery bypass grafting can be used if the damage to the vessel wall is long, which avoids missing the injury with postoperative embolism as a result. We documented the case of a patient who suffered a thrombosis immediately after direct anastomosis end-to-end surgery and who was then re-operated on using grafting with saphenous vein. The cause of complication may be not completely removement of the damaged segment, short length and tension of vessel, may also be due to the failed technique or the inadequate anticoagulation (Figure 7).

### 4.5. Limitations

Several limitations need to be noted in this article. First of all, in operating on these patients, it was most important to us to restore vascular circulation, the recovery of bone anatomy being secondary. Hence, although there is no anatomical perfection, the bones have been less displaced. Additionally, while there was a follow-up in this study, the period for evaluating patient outcomes was within one month following surgery. There are two main reasons for this which should be acknowledged. Firstly, the main purpose in our study was to focus on treating early-stage vascular damage. Secondly, because COVID-19 lockdowns have been continuously imposed in Vietnam and out-of-province patients are those who have been most affected by this, the re-examination of patients after surgery has been very difficult in our institution. Therefore, longer-term outcomes have not been evaluated in this study.

## 5. Conclusions

The majority of supracondylar humerus fractures with brachial artery injuries did not present with signs and symptoms of critical limb ischemia similar to peripheral vascular injuries in lower extremity; therefore, emergency management was not required in all cases. Diagnosis and treatment for pediatric supracondylar humerus fractures with vascular injury is a difficult and time-consuming procedure, especially in cases of transverse fractures. Two-thirds of patients who underwent surgery had no physical damage to the blood vessel wall and lumen. As a result of this study, we propose to apply a unique protocol in the management of pediatric supracondylar humerus fractures with brachial artery injuries with the aim of shortening treatment duration and minimizing the performance of unnecessary procedures and surgical treatments.

## Figures and Tables

**Table 1:** Clinical characteristics at admission.

Characteristic	Patient (*N* = 50)
Gender, no. (%)	
Male	36 (72.0)
Female	14 (28.0)
Age, years	
Mean (years)	5.85
Min–Max (years)	1.5–14
Time interval between the beginning of trauma and arrival to the first health facility (hours)	
Mean (hours)	12
Min–Max (hours)	1–120
Time interval between the beginning of trauma and arrival to the operating room (hours)	
Mean (hours)	52.8
Min–Max (hours)	4–168
Mechanism of injury, no. (%)	
High energy trauma	49 (98.0%)
Other *	1 (2.0%)
Injured side, no. (%)	
Right arm	19 (38.0%)
Left arm	31 (62.0%)
Gartland classification of fractures, no. (%)	
Type II	4 (8.0%)
Type III	46 (92.0%)
Types of bone fractures, no. (%)	
Closed fracture	49 (98.0%)
Open fracture	1 (2.0%)
Ischemia in the upper extremities, no. (%)	
Cold limb	11 (22.0%)
Warm limb	39 (78.0%)
Skin color in hand, no. (%)	
Pink hand	49 (98.0%)
Purple hand	1 (2.0%)

* This involved the arm being rammed into by a buffalo.

**Table 2:** Brachial artery injuries and flow velocity around the fracture site with Doppler sonography classification, before and after casting.

	Before Cast (*N* = 50)	After Cast (*N* = 50)
Brachial artery injuries, no. (%)		
No assessment	43 (86%)	45 (90%)
Thrombosis	1 (2%)	0 (0%)
Contusion	2 (4%)	3 (6%)
Vasospasm	4 (8%)	2 (4%)
Flow velocity under the fracture site (m/s), mean (SD)	16 (8.5)	18.2 (9.7)
Doppler waveforms		
Monophasic flow	22 (44%)	25 (50%)
Biphasic flow	28 (56%)	21 (42%)
Triphasic flow	0 (0%)	4 (8%)

**Table 3:** Frequency of treatment methods with age group and length of lesions on multislice CT-A.

	Surgical Treatment (*N* = 36)	Conservative Treatment (*N* = 14)
Age group (yrs), Frequency (%)		
<3 years	2 (5.56)	1 (7.14)
3–<13 years	32 (88.89)	13 (92.86)
≥13 years	2 (5.56)	0 (0.00)
Patient age, mean (SD)	6.28 (2.87)	4.75 (2.06)
	**Surgical Treatment** **(*N* = 21)**	**Conservative Treatment** **(*N* = 7)**
Length of lesions on multislice CT-A		
Lesions < 5 mm	1 (16.7%)	5 (83.3%)
Lesions ≥ 5 mm	20 (90.9%)	2 (9.1%)

**Table 4:** Association of intraoperative vascular injury with age group, lesions on multislice CT, preoperative Doppler spectrum analysis and brachial artery graft in pediatric patients undergoing surgical treatment.

	Vasospasm	Vascular Contusion/Thrombosis
Age group (years), no. (%)		
<3 years	2 (100%)	0 (0%)
3–<13 years	22 (68.8%)	10 (31.2%)
≥13 years	0 (0%)	2 (100%)
Length of lesions on multislice CT-A, no. (%)		
Lesions < 5 mm	1 (6.7)	0 (0.0)
Lesions ≥ 5 mm	14 (93.3)	7 (100.0)
Doppler waveforms, no. (%)		
Monophasic flow	17 (71.4)	7 (58.3)
Biphasic flow	7 (28.6)	5 (41.7)
Brachial artery graft, no. (%)		
Blockade	11 (100%)	0 (0%)
Angioplasty	13 (100%)	0 (0%)
Anastomosis end-to-end or using graft	0 (0%)	12 (100%)

**Table 5:** Postoperative results and one-month re-examination (*N* = 36).

Postoperative Results	Patient
Embolism immediately after surgery, no. (%)	1 (2.8)
Superficial infection immediately after surgery, no. (%)	2 (5.5)
Temporary loss of sensation around an incision one month after surgery, no. (%)	2 (5.5)

## Data Availability

All the data that support the findings of this study are available from the corresponding author on reasonable request. Requests for access to these data should be made to Tu Ngoc Vu (Email: vungoctu@hmu.edu.vn).
